# Parental Perspectives and Experiences in Relation to Lifestyle-Related Practices in the First Two Years of a Child’s Life: A Qualitative Study in a Disadvantaged Neighborhood in The Netherlands

**DOI:** 10.3390/ijerph17165838

**Published:** 2020-08-12

**Authors:** Gülcan Bektas, Femke Boelsma, Vivianne E. Baur, Jacob C. Seidell, S. Coosje Dijkstra

**Affiliations:** 1 Faculty of Science, Vrije Universiteit Amsterdam, Amsterdam Public Health Research Institute, De Boelelaan 1105, 1081 HV Amsterdam, The Netherlands; f.boelsma@vu.nl (F.B.); j.c.seidell@vu.nl (J.C.S.); 2 Department of Care Ethics, University of Humanistic Studies, Kromme Nieuwegracht 29, 3512 HD Utrecht, The Netherlands; v.baur@uvh.nl

**Keywords:** parental practices, lifestyle-related behavior, sociocultural influence, qualitative study, infant

## Abstract

The first two years of a child’s life are a critical period in preventing several lifestyle-related health problems. A qualitative study was conducted to explore parental experiences and perspectives in relation to lifestyle-related child-rearing practices in order to minimize risk factors at an early stage. Data were collected through interviews (*n* = 25) and focus groups (*n* = 4) with parents of children aged 0–2 years, in a disadvantaged neighborhood in Amsterdam, the Netherlands. Results showed that parents were often uncertain about a number of lifestyle-related practices. Ambiguity also appeared regarding the parents’ intentions to engage in certain practices and what they were able to achieve in everyday life. In addition, parents experienced strong sociocultural influences from their family, which interfered with their ability to make their own decisions on lifestyle-related practices. Parents also expressed a need for peer-support and confirmation of their practices. Future studies should focus on supporting parents in their parental practices during the first two years of their child’s life. Any such study should take into account the specific sociocultural context accompanying lifestyle-related parental practices.

## 1. Introduction

The first two years of a child’s life are a crucial period, during which the foundations for a healthy growth and development are established [1,2]. Several studies have indicated that this period is essential in the prevention of lifestyle-related health problems later in life, such as obesity, cardiovascular diseases and type 2 diabetes [3,4,5,6]. There is clear evidence that these lifestyle-related problems are largely caused by unhealthy dietary behaviors, low levels of physical activity, high levels of screen time, and inadequate sleep among children [7,8,9], and it is known that these unhealthy lifestyle behaviors are more prevalent among children of families with a lower socioeconomic position (SEP) and certain disadvantaged ethnic groups [10,11,12,13]. The reasons for the differences in these lifestyle-related behaviors among children from these backgrounds are complex, and are likely to be related to differences in genetic susceptibility, cultural identity, family composition, lifestyle preferences, and the physical and socioeconomic environment [14,15,16].

Since most lifestyle-related behaviors develop in the first two years of life and are shaped by parents, one of the most important factors influencing lifestyle-related behaviors in children is parental practices [17,18,19,20]. These practices are the specific behaviors that parents engage in as they raise their children and refer to their choices in behavioral areas such as the use of schedules, rules, restrictions, or rewards [21,22]. Some parental practices during the first two years of a child’s life may increase or decrease the risk of developing health problems [23,24]. For instance, a relationship has been repeatedly shown between developing childhood obesity and the food-related practices of parents, such as the early introduction, frequency, and portion size of solid food [8,25].

Several studies have shown that socioeconomic and ethnic inequalities in lifestyle-related problems during the first two years of a child’s life can partly explain differences in parental practices [12,26,27,28]. For example, a study among the Hispanic population in the United States showed that unhealthy dietary behaviors such as early introduction of solid foods and the provision of unconventional food products are more common in children with a migration background [29]. Similar results were found in the Netherlands, where a study showed that mothers who breastfed for shorter duration are more likely to introduce solid food early on [30]. In addition, another study in the Netherlands reported that 20.2% of the Dutch infants were consuming sweet beverages daily, which was associated with lower maternal educational level and absence or shorter duration of breastfeeding [31].

However, most studies investigating differences in parental practices are quantitative and do not provide adequate insights to unravel the underlying parental experiences, perspectives and associated factors that play a role in practices that can lead to unhealthy lifestyle behaviors in children.

The overall aim of this qualitative study is therefore to explore the experiences and perspectives of parents with regard to their lifestyle-related practices and associated factors in the first two years of a child’s life. The study took place in a disadvantaged and ethnically diverse neighborhood in Amsterdam, the Netherlands. The results are likely to be useful in the development of new intervention strategies that seek to promote a healthier lifestyle for young children.

## 2. Materials and Methods

### 2.1. Study Design

This study forms part of the larger Food4Smiles project, which has been set up to examine the healthy growth and development of children in a disadvantaged neighborhood in Amsterdam, the Netherlands, during the first thousand days. In order to gain insight into the perspectives and experiences of parental practices and associated factors, parents with children aged 0–2 years were the main focus. The study took place in one of the ‘focus districts’ identified by the local government, with regard to the prevention of multiple health problems (e.g., childhood obesity, depression, loneliness) as well as socioeconomic problems (e.g., poverty, low literacy, unemployment). The neighborhood is considered to be very culturally diverse, which is attributable to a high level of migrants. Fifty-two percent of the population are of non-Western descent, and the population includes more families and children than other neighborhoods in Amsterdam. Dutch, Moroccans and Turks are the largest ethnic groups in the neighborhood (34.3%, 20.9% and 12.9%, respectively). Of the people in the neighborhood, 25% are under the age of 17, and 20% of them are growing up in a poor household (an income up to 110% of the Dutch minimum standard and capital below the social welfare limit). The prevalence of overweight in the neighborhood is high, with, for example, 47% of adults being overweight or obese, 28% of the 10-year-olds, and 11.2% of the 3-year-olds. The prevalence of childhood obesity is highest among children of Turkish and Moroccan descent—29.8% and 21.4%, respectively [32,33].

Because of the exploratory nature of the study, we chose a qualitative design [34]. First, we conducted semi-structured interviews with the respondents to obtain more detailed information about the individual experiences and perspectives of parents with regard to parental practices associated with lifestyle-related behaviors such as feeding, sleeping, screen time, and daily struggles, and also about the health and weight status of their child, the type of perceived support and the experienced barriers/facilitators in their social environment (for interview guide, see Appendix A). Parental practices were defined as specific behaviors through which parents fulfil their parental duties and include parental beliefs about the use of schedules or restrictions concerning food, involvement in children’s activities, setting rules about screen time and bedtime, and the influence of contextual factors such as grandparents’ parenting practices [35,36]. The interviews were conducted by the principal researcher (GB) who was a second-generation immigrant of Turkish descent. She was also born and raised in the neighborhood under study, and is still living there. Subsequently, focus groups were conducted to verify the findings from the interviews and to collect richer data on specific topics. The focus groups addressed in-depth topics about the role of the father and the extended family (e.g., grandparents) in the daily practices and taking care of (grand) children, cultural influences, beliefs about childhood obesity, and maternal instinct. In the focus groups, the topics were discussed using cards with statements or questions about the various topics (see Appendix B). The cards were scattered on the table and respondents were encouraged to pick up a card from the table at random and start talking about the topic. The focus groups were led by two researchers (GB and FB) in order to minimize researcher bias. The presented results in this article were based on both the interviews and the focus groups.

The Medical Ethical Committee of Amsterdam UMC (VUmc location) concluded that this study was not subject to the Medical Research (Human Subjects) Act (WMO).

### 2.2. Data Collection

Parents who were expecting a child and/or parents with infants aged 0–2 years were recruited in the period from May 2018 to July 2018. In order to represent all relevant aspects (e.g., gender, education level, ethnicity) in the study, the main selection criterion was a maximum variation in respondents in the neighborhood. Recruitment of respondents took place through different channels, such as youth health care centers, approaching the target group in neighborhood playgrounds or parks, and publicizing the study through existing parents’ app groups. Respondents had to be able to understand and speak Dutch, English or Turkish, as these were the three languages spoken by the principal interviewer. With the agreement of the respondents, the interviews were conducted at their homes, or at an alternative location in the neighborhood if that was more convenient. The interviews were recorded after consent and were transcribed verbatim. The interviews varied in length from 60 to 130 min and notes were made of any unrecorded information (e.g., additional information or explanation given after the recording had ended). The interviews were primarily conducted in Dutch, while the interviews with Turkish-speaking respondents were partially (*n* = 5) or completely (*n* = 1) conducted in Turkish. The sections in Turkish were transcribed verbatim and then translated into Dutch by the principal researcher, who conducted the interviews. Once the interviews had been completed, four additional parent focus groups were formed to discuss a range of topics. The focus group sessions were recorded and varied in length from 60 to 90 min. The discussions were primarily conducted in Dutch, with the exception of one focus group, which was partially in English. Sections in English were transcribed verbatim. The following data were collected for each participant at the end of each interview and/or focus group: gender, age, level of education, ethnicity, family structure, number and age of children.

All respondents received a letter informing them about the study, and all of them signed a written statement of informed consent. Transcripts were processed anonymously; the names and other identifying features of the respondents were coded. The characteristics of the respondents were made available, but could not be attributed to any individual.

### 2.3. Respondents

In total, 39 people were approached for an interview, 25 of whom were included in the study. Reasons for ineligibility or declining to take part were not living in the target district (*n* = 4), having children older than 2 years (*n* = 3), not interested (*n* = 2), unreachable by phone (*n* = 3), and unable to schedule the interview due to holiday (*n* = 2). A total of 35 parents were invited to take part in the focus groups: 21 parents agreed to participate, 6 declined, and 8 were unavailable at the time of the focus group. All respondents in the focus groups were mothers. The focus groups included both mothers who had previously participated in the personal interview (*n* = 8) and new mothers (*n* = 13). Consequently, our study included mainly women. Educational background was defined in terms of the highest level achieved and was classified as follows: no education/primary education (low); lower secondary education/higher secondary education (middle); higher vocational college/university (high). Ethnic background was established with reference to country of birth: either the respondent’s own country of birth or the country of birth of one of their parents. Most of the respondents were born and raised in the Netherlands (*n* = 27), 3 respondents were not born in the Netherlands and those years of residence in the Netherlands were 7, 10 and 21 years, respectively. The country of birth or years of residence in the Netherlands of 8 respondents were unknown. Table 1 presents the characteristics of the respondents (see Supplementary Materials).

### 2.4. Data Analysis and Quality Procedures

All transcriptions were summarized and sent to the respondent for a member check [37]. Respondents were asked whether the views they expressed had been interpreted correctly by the researcher and if they thought the summary was an accurate reflection of the interview or discussion. The principal researcher (GB) coded the data using ATLAS.ti [38] and identified recurring themes in the interviews. The main categories of the code tree and initial codes were deductively determined from the interview guide. A thematic content analysis was used in order to be as open as possible to the experiences and perspectives of the respondents. Subsequently, key fragments were identified on the basis of a keyword or several keywords. To increase reliability and ensure uniform coding, a second researcher checked the transcripts and labelled them independently. Two researchers (GB and FB) both coded the first 10 interviews independently and then compared their coding to check for discrepancies in their analyses. Consensus was reached on most topics; some topics were further explored to find consensus. A discussion on the labels then took place in order to identify important similarities and resolve differences. Next, the labels were redefined and clustered into main themes and subthemes, and all transcripts were analyzed using these labels by the principal researcher. Finally, after the axial coding process, the final themes were established and all interviews were coded on the basis of these final themes.

## 3. Results

Four main themes emerged from the experiences and perspectives shared by parents about their lifestyle-related practices in relation to their child. First, parents experience uncertainties about satiety, weight, and healthy growth of their child. Second, parents express ambiguity about their intentions to engage in practices and what they are able to achieve in everyday life. Third, parents experience a strong sociocultural influence from their family, impeding their ability to make their own decisions with regard to lifestyle-related practices. Finally, parents experience parenting as a relational practice and look for support and confirmation.

### 3.1. Parents Experience Uncertainties about Satiety, Weight, and Healthy Growth of Their Child

When asked to describe ‘a healthy child’, parents often said that a healthy child is a child who eats well. In all of the interviews, parents associated healthy infants with being well-fed and saw eating well as an important factor in growing up healthy. Many parents were more concerned about their child being underweight than overweight, and this seemed to relate to a fear of the child becoming malnourished. Consequently, parents seemed to prefer their child to eat more than necessary rather than less. Several mothers who were breastfeeding were uncertain whether the breastfeeding was sufficient to satisfy their child and were worried whether their milk supply was adequate. Some mothers felt that their insufficient milk production left their infant hungry and said they tried to compensate for this with extra bottle feeds or by switching to bottle feeds altogether.

“Sometimes I give him an extra bottle feed, because I don’t think my milk production is enough. He finished one breast and then took the other, but he is still…didn’t have enough. I think it isn’t enough for him, so I give him a bottle feed in addition to the breastfeeding. Especially at night, because I think I have less milk production in the evening.”

(Mother of a 5-month-old son)

Furthermore, most parents did not seem to worry about excessive weight gain during infancy as a risk factor in the development of childhood obesity. Being overweight was only seen as a problem in older children. In addition, all parents were convinced that children would lose weight once they started to become more active, so they did not see it as problematic for an infant to have a little extra weight.

“Well, I think it doesn’t matter if babies are chubby or overweight, because that’s something they will outgrow. When they start to move around you often see the baby fat disappear. So I worry less about a chubby baby.”

(Mother of an 18-month-old daughter)

Due to the assumption that weight gain during infancy would be compensated when a child became more physically active, most parents did not see the need to manage an infant’s weight gain. Some parents had been told by healthcare professionals that their children were at risk of being overweight/obese, but some thought that the healthcare professionals were exaggerating or misrepresenting the situation. In their opinion, the healthcare professionals did not measure consistently and did not take personal factors such as genetics or cultural background into account. Most of the respondents expressed the view that a chubby infant was acceptable.

“They [babies] also need to have some reserves, because when they are sick they lose weight very quickly. So I think it can only be a good thing if a baby is chubby. I see that as a kind of reserve for periods of weight loss. I wonder why it should be bad or unhealthy for a baby to be chubby?”

(Mother of a 3-month-old daughter)

### 3.2. Parents Express Ambiguity in Relation to Their Intentions to Engage in Practices and What They Are Able to Achieve in Everyday Life

Most parents reported that they continuously thought about their parenting practices and considered the effect they were having on their child’s health. Parents sometimes expressed differences between their intentions and values, and their actual practices in daily life. For instance, they expressed the belief that physical activity is important to a child’s health and development, but in practice they had little time to encourage or promote physical activity.

“I often try to walk and move with him, because I think it’s healthy. But it’s second nature for us [to take the baby with the stroller, ed.] so it actually happens unconsciously. I think he’s still too young to walk with me. […] And it’s easier to take him in the stroller when I’m busy and don’t have much time.”

(Mother of 2 sons, 8 years old and 11 months old)

In addition, most parents said they found it difficult to promote physical activity, since they did not always know what to do with their infants and were sometimes unsure about what activities were age-appropriate. Parents were also less worried about encouraging their child to engage in more physical activity, expressing the belief that children are naturally inclined to be physically active. Most parents assumed that spontaneous movement is sufficient activity for a child aged 0-2 years, and did not relate levels of physical activity during infancy to health status in later life. Parents expressed most ambiguity in relation to screen use. Almost all parents stated that they wanted to avoid screen use or limit the amount of screen time for their children (television, tablet, smartphone) because they had read or had been informed that excessive screen time can have a negative effect on a child’s development. Parents with more than one child recognized that having older siblings leads to earlier screen use in infants, because the older children are allowed to use screens and the younger children copy their behavior. Even though parents indicated that they wanted to limit screen time for their young children, they said they regularly used television, tablet or smartphone as a way to keep their child occupied—for example, when they needed to do household chores. Some parents also said they had started using screens (smartphone, television) to distract their child when they were feeding them, because they were more inclined to eat when they were distracted. Many parents also believed that their infants should be familiar with using screens from an early age, so that they can develop their digital skills early on. Parents also reported that they considered television, tablet or smartphone viewing to be educational and an important element of their infant’s visual, cognitive, and social development. A considerable proportion of respondents said their infant was allowed about 30–60 min of screen time per day for educational purposes.

“I give him the iPad when I sleep, so that he can watch cartoons in Arabic. I want him to learn both Arabic and Dutch.”

(Mother of two sons, 2 years old and 4 months old)

### 3.3. Parents Experience a Strong Sociocultural Influence from Their Family, Impeding Their Ability to Make Their Own Decisions on Lifestyle-Related Practices

All parents acknowledged that their extended family has a considerable influence on daily family life and the lifestyle-related practices with regard to their children. Grandparents in particular were mentioned as a source of support and advice for parents. Most parents said they valued the advice and recommendations on parenting practices given by members of their extended family, who were often very experienced in raising children. In a number of families, the grandparents took care of the children a few days a week when the parents were working. Some parents observed that the grandparents’ practices differed from their own, and sometimes parents disagreed with these practices. For example, several parents said that family members wanted to give their infant food and drinks that were not recommended for a child that age, such as sugar-sweetened beverages (fruit juice, lemonade), salty and spicy meat or chicken, or fruit yoghurt with added sugar. According to the parents, the grandparents were inclined to give the infant these products because they believed it would make the child more robust and help them develop appropriate taste preferences. Some parents expressed their disagreement with the grandparents’ practices but found it difficult to discuss this with their family members. The parents remarked that the practices and beliefs of their family members were deeply rooted in their culture and were therefore difficult to question.

“She [mother-in-law] gave him honey, because he had a bit of a cold. I said, ‘Don’t give him that, it can be life threatening.’ She says, ‘But I have always given my children that!’ She really is a bit more easy-going about that kind of thing. The Moroccan community thinks that everything with sugar is good.”

(Mother of 2 sons, 8 years old and 11 months old)

Parents also said it was difficult to discuss certain practices because they did not want to offend their own parents, and they understood that grandparents want to indulge their grandchildren. Parents who received support from their own parents or parents-in-law in caring for or nurturing their children found it particularly hard to correct their family members. Some mothers expressed the feeling that they should be grateful for the extra help they received from family members and were therefore hesitant to complain about any practices they disagreed with.

“Well, my mother-in-law takes care of him two-and-a-half days a week. And when he’s there he gets food all the time. I have to let that pass and just go with the flow. I am really grateful that she takes care of him.”

(Mother of 2 sons, 8 years old and 11 months old)

When parents do express their disagreement with the grandparents, they often find that the grandparents do not listen to them and continue their behavior.

“She [mother-in-law] keeps saying, ‘My sons grew up eating tomato paste, salt and all these spices, and they are healthy.’ And I say, ‘No, that is no longer approved. The guidelines are different.’ But she keeps insisting.”

(Mother of a 10-month-old son)

Grandparents also appeared to have an influence on the children’s use of screens such as television or smartphone, and did not put any limit on screen time, according to the parents. A few mothers said the grandparents particularly used screens to encourage the children to eat well, and one of the mothers said it was her mother who taught her daughter to eat while being distracted by the smartphone.

“My mother probably taught her to use the smartphone. Because she handed her the phone so that she would eat and forced her to open her mouth. It was just a way of distracting her.”

(Mother of 3 children, a 7-year-old son and daughters aged 18 months and 4 months)

Most parents said that grandparents seemed to prefer chubbier infants, because they associated bigger infants with fewer ailments and better health. A few parents felt pressure from the grandparents to have chubbier infants, and some mentioned that the grandparents sometimes criticized how they fed their children and felt that the children were not being fed enough.

“I also wanted them to be a bit fatter, but I don’t make an issue about it. But my mother always says, ‘Look at your friend’s child–she looks nice with those chubby cheeks.’”

(Mother of 2 sons, 5 years old and 18 months old)

Some parents also reflected that the preference for chubbier infants was rooted in their culture and that they felt some pressure from their social environment to have chubbier children.

“For example, my children are thin. Thin… I mean, I think they are a normal weight but people are saying: ‘Aren’t they eating anything? They are so thin!’ Older people especially say that kind of thing.”

(Mother of 3 children–sons aged 6 and 4, an 18-month-old daughter–and pregnant with her fourth child)

Parents also remarked that cultural factors and environmental aspects could also be counterproductive in practices relating to sleep. Some mothers explained that it is common in their culture for children to go to bed late. In particular, visits from family or friends, who sometimes drop by unexpectedly, can have a negative influence on the children’s bedtimes, because children can find it hard to sleep due to the people or noise around them.

“In our culture, it’s usual to drop in on people unexpectedly. We visit each other often and stay until late in the evening. Many families find this difficult to deal with. The baby’s sleeping times get disrupted. The children often go to sleep late if we’ve had visitors.”

(Mother of 3 children–sons aged 6 and 4, an 18-month-old daughter–and pregnant with her fourth child)

### 3.4. Parents Experience Parenting as a Relational Practice and Are Looking for Support and Confirmation

Most parents said that they would appreciate more practical and specific advice on the following themes in order to stimulate the healthy development of their child: (breast) feeding and frequency, when to start introducing solid foods and how to prepare and conserve these foods, suitable physical activities for children aged 0–2 years, handling and recognizing crying cues, sleep routines, and insights into normal child development. Parents do not want to receive general information, but they are interested in receiving strategies and tips that they can implement in their daily practice. Parents expect child healthcare staff to provide them with factual information and practical tips, but some parents felt that the information they received was not specific or practical enough.

“She [member of staff at Child Health Center] told me to start him on solid food, but she didn’t tell me which vegetables, or how much to give him or how I should prepare them. That’s all new for me. The only thing she said was: ‘You can start now.’”

(Mother of a 4-month-old son)

Additionally, all of the parents said that they valued contact with their peers: parents with children in similar age categories. According to the parents, peers are a valuable source of information, because they have experienced similar situations and can provide helpful and practical support. Parents also expressed the need to meet more peers with young children, for social support and to exchange experiences. Some parents felt isolated because they were at home most of the time after their baby was born. Parents felt there was little opportunity in their neighborhood for parents with children aged 0–2 years to do something outside the home or to take part in activities and meet each other. The majority of the parents therefore suggested facilitating community parent-encounter groups, where they would be able to find peer support and exchange information informally. They also believed that these groups could foster mutual understanding, and provide children with the opportunity to socialize and learn to play with other children.

## 4. Discussion

This qualitative study provides insights into the experiences and perspectives of parents with regard to lifestyle-related practices and associated factors in the first two years of their children’s lives in a disadvantaged neighborhood in the Netherlands. The key finding of this study is that parents experience uncertainties about their lifestyle-related practices, occasionally resulting in unfavorable practices such as overfeeding their child. The respondents expressed ambiguity with regard to their intentions to engage in parenting practices and what they were able to achieve in daily life. These parents were continuously assessing the value and the effect of their practices on their children’s health and experienced difficulties in putting their intentions into action. This seemed to be related to the fact that parents did not immediately see the impact on their children’s health of lifestyle-related practices such as overfeeding or screen use in early childhood and believed that most of these things could be compensated in later life. Parents also experienced strong sociocultural influences from their wider family, which not only could make them uncertain about their practices but also impeded them in their ability to make their own decisions about parenting practices. Parents also expressed a need for peer support and confirmation of their practices.

Our study found that the parents were particularly concerned about their feeding practices and uncertain about the interpretation of hunger and satiety, the weight and growth of their child, and that they tend to underestimate the risk of excessive weight gain during infancy. Similar results were found in a previous study among Hispanic mothers in the United States, which reported that mothers had concerns around the food intake and satiety of their children and believed that they should feed their child until they were full [29]. In addition, several studies have shown that parents underestimate their children’s weight and do not associate body weight and healthy behavioral practices in the early years with potential problems in the future [24,39,40,41,42,43]. This could explain unhealthy parental practices such as overfeeding in our study, since parents fear malnourishment and believe that children should build up energy reserves that they can draw on during difficult periods (e.g., illness) or when they start becoming more mobile and physically active.

Parents assumed that their children’s spontaneous inclination to move around constituted sufficient activity for their age and believed that this was enough to ensure healthy development. Previous studies which examined the physical activity of preschool-aged children also reported that parents believed that children were naturally inclined to be physically active and that this belief made them less engaged in promoting physical activity in their children [44,45,46]. However, our study found that while parents wanted to be engaged in or promote physical activity in their children, they did not always know what activities were appropriate to the age group. Another consideration was striking a balance between the time available to engage in physical activity with their children and performing other tasks. Parents’ lack of time and lack of awareness of the importance of physical activity during infancy for later life stages resulted in less support for physical activity. In addition, parents were also less worried about sleep, since they did not associate curtailed sleep with future health problems. This finding corresponds with earlier results of a qualitative study where parents did not recognize curtailed sleep as a risk factor in childhood obesity [29].

Although parents spoke of the importance of limiting screen time for their children, most of them did not impose limits on the screen use of their younger children. In practice, it was difficult for parents to limit screen use, because this was an easy way to keep children occupied or distract them so that they would eat better. Parents also stated a belief that screen viewing contributes to a child’s cognitive development and that it is acceptable for parents to allow their children to use a screen when they are busy, while most of them also expressed a positive opinion about the educational value of screen time [39].

In line with earlier research, our study also found that grandparents play an important role in the care of young children and have a significant influence on the parents’ lifestyle-related practices [47,48]. Several studies have described that the extended family exerts an influence on the eating habits of infants, such as the introduction and the amount of solid food, supporting parents in finding an approach to healthy lifestyle behaviors for their children [27,49,50,51,52]. Our study also found that grandparents are influential in the eating habits of children. However, we observed that the extended family does not always contribute to healthy lifestyle behaviors and that their influence can also lead to feelings of uncertainty in parents with regard to what is healthy or unhealthy, resulting in a tendency among parents to overfeed their children, a practice which often directly contradicts the recommendations given by child healthcare professionals. This may explain the need expressed by parents for more practical, concrete, and tailored information to help them judge what they should be feeding their child and how, since there appears to be a relationship between their uncertainties as parents and their lack of parenting knowledge or skills. For example, parents said that while child healthcare staff told them when to introduce solid foods, they were not given any practical advice about what to feed their children or how to prepare it. This lack of information led parents to experience even greater uncertainties, which in turn made them more likely to follow the advice offered by their social environment. The study also revealed a “normative expectation” among grandparents that infants should be chubby, which is confirmed by previous studies that reported a preference among grandparents for a chubbier infant [47]. However, the added value of our study is that it provides insights into how the extended family influences parental practices, the strong relationship between this influence and sociocultural norms and values (including those that govern family dynamics), and how this influence can be experienced as pressure by parents.

The sociocultural values, boundaries, and family dynamics are all important aspects of our analysis and shape the roles, identities, responsibilities, and behaviors of parents and their subsequent parental practices. This can be explained by two mechanisms: the task-responsibilities of parents and the social demand that a child should correspond with the normative perspective on what constitutes a healthy child. Our findings show that a considerable proportion of the parents felt a personal responsibility to comply with sociocultural expectations. This could be explained by a relational perspective on practices or responsibilities [53] and offers an alternative to the theoretical-judicial model of moral theorizing that places morality in the realm of unsituated, individualistic, rationalist, and intellectualist knowledge. The alternative is an expressive-collaborative model based on the idea that people understand themselves to be bearers of particular identities and actors in various relationships that are defined by certain values. The way someone acts, or the role that someone fulfills, does not therefore stem from abstract principles and rationalist reasoning, but is formed against the collaboratively constructed and deeply situated backgrounds of understandings about what people are supposed to do and be [53]. Examining our findings through this perspective, we see that parents’ moral understandings of what good parenting (as a value) means are formed in relational practices in which diverse identities and relationships play a role and sometimes collide. Our study found that a chubbier infant is more appreciated by the ethnic and cultural community of which these parents are a part, and that parents felt pressure to have chubbier infants. It is well-known that some ethnic minority groups have different perceptions about what constitutes a healthy child, and that in some social groups thinness can be seen as a sign of vulnerability [24]. Even though parents may not always agree with the normative assumptions of their community, social pressure often makes them conform to the assumptions, values, and preferences of the dominant culture. For instance, some mothers in our study stated that they were criticized by their community when their child was seen as being thin according to the perceptions of their culture. Community or family members asked them whether they were giving their child enough to eat and gave unsolicited advice about how to feed their child properly, making them feel uncertain or guilty in the process. This led to a desire among the parents to fulfill their “responsibility” to conform to the expectations of their community in order to be recognized as a good parent [54]. Normative social assumptions can therefore shape the lifestyle-related practices of parents in the first years of a child’s life, and these practices are also further embedded in the existing distributions of power in the familial system. At the same time, parents’ values with regard to healthy child-raising are also shaped by other societal influences, such as child healthcare services and the Dutch culture in which they operate. This can lead to conflicts with their familial system and make parents feel confused or uncertain about their own practices.

Parents need to rely on grandparents for support, which puts grandparents in a powerful position [55]. There is an interfamilial component to support in child-rearing, which forces parents into a dependent position [27,52]. The majority of our respondents were second generation migrants (55%), which means that they are grown up in pluralistic society and might have adopted cognitions and practices of the Dutch culture. Although most parents said they did not agree with the unhealthy practices of grandparents, such as giving honey to an infant or giving children food continuously throughout the day, they were hesitant to complain about them due to the sociocultural norms and values of their cultural background, not least the tenet that parents, parents-in-law and older members of society in general should be treated with respect. For instance, a mother explained how her child’s grandmother distracted the child with a screen at feeding time, but she did not dare to complain about it, because the grandmother provided her with support and she did not want her to think she was being ungrateful. Furthermore, the grandmother has lived longer and has raised children of her own, and the social environment dictates that the mother should respect the knowledge and experience of an older woman. Other empirical findings show that in some cultures older people are given a social status on the basis of their age and experience [52]. The sociocultural background expects parents to be respectful towards the older generation, in particular their in-laws, and that they should be grateful for the help they receive. The sociocultural networks in which the parents operate persuade them to be less resilient towards the grandparents on both sides of the family and explains why they display a passive attitude. Faced with the prospect of feeling guilty if they go against these sociocultural expectations or demands, parents tend to surrender to them. According to Bowen, this behavior prevents conflict and ensures more stability in the familial system [55].

This study has several strengths and limitations. One strength is the background of one of the researchers who was born and raised in the neighborhood. This created a climate in which the respondents felt more comfortable and secure. Her deeper understanding of the community had a positive influence on the level of openness shown by the respondents. At the same time, this had the disadvantage of respondents expecting that the interviewer would readily understand them, which made them less inclined to elaborate on some issues. As a result, greater emphasis was placed on certain concepts such as “family dynamics” in the focus groups in order to gain a deeper insight. The focus groups were led by two researchers in order to minimize researcher bias. The researchers observed that the recording of the interviews and group discussions was a barrier for some respondents in terms of feeling able to speak freely. In some cases, respondents became more outspoken after the recorder was turned off, with parents then feeling more comfortable and revealing more details about the obstacles and social pressures they had encountered. To capture these contributions, notes were made after the recorder had been turned off. Furthermore, the principal researcher spoke three languages (Dutch, English and Turkish), which enabled the Turkish respondents to express themselves in Turkish if they felt themselves more comfortable in that language. This could mean that we had an underrepresentation of parents who do not speak any of these languages (e.g., Moroccan). The present study also included significantly more mothers than fathers, since fathers were less willing to participate. Fathers are known to exert a considerable influence on the food intake, screen time, and physical activity of young children [29,56]. There is, for instance, evidence that the father’s dietary intake could predict a child’s dietary intake and that fathers place less limits on snacks or are less likely to ensure the consumption of a variety of foods and daily access to fruits and vegetables [57,58]. This could have an influence on the practices of a mother and achieving a greater insight into the perspectives of both fathers and mothers is needed. In the current study, recruiting fathers through child-centered venues was not an effective strategy, since it was difficult to find fathers that were willing to participate. Future studies should try to include more fathers and use specific techniques to be more effective in this. The use of a male interviewer could help in order to increase fathers’ willingness to participate and engage them in future studies. Despite the fact that this study was conducted in a disadvantaged neighborhood, only half of the respondents had a low or medium level of education. A considerable proportion of the respondents were highly educated, and therefore, the results of this study may not be representative of the population of the district as a whole.

The current study was focused on lifestyle-related parental practices and their associated factors, since lifestyle-related parental practices in the early lifespan are complex and influenced by many factors such as environmental, social and economic factors [12]. During the interviews and focus groups, the researchers deepened the answers of the parents by asking questions in order to find underlying mechanisms (e.g., the role of the social environment or cultural influences). However, we are aware of the fact that we were not able to address all associated factors, such as the role of poverty or more private affairs. Future studies, with other study designs, should therefore focus on more in-depth insights that are associated with the lifestyle-related practices of parents.

## 5. Conclusions

In conclusion, this study shows that parents are uncertain about their lifestyle-related practices in the first two years of their children’s lives. They express ambiguity with regard to their lifestyle-related parenting intentions and how they put these intentions into practice. Furthermore, the study highlights the strong influence that the extended family exerts on parenting practices. Given the crucial role that the social environment plays in parental practices, fueled by social expectations, it is important to create a supportive climate among the people who play a key role in the lives of the parents of young children. Any solutions should therefore take into account the role of grandparents and the specific sociocultural context in which parental practices take place. Additionally, parents highlighted the potential of facilitating community peer groups as a valuable resource for obtaining information in an informal setting, and suggested that this could fulfill the need among parents for more practical and specific information on all aspects of lifestyle-related practices in line with their own perceptions.

We recommend that future studies and interventions focus on the support of parents, enabling them to improve their parental practices, abilities, and skills in the first two years of their children’s lives. Interventions should provide opportunities to discuss the underlying and sometimes conflicting values and beliefs about what it means to raise a healthy child and what good parenting entails. Getting parents involved in collaborative and dialogical learning processes in their direct social environment is important, in order to create spaces in which parents and their social environment can share in each other’s narratives and find ways to deal with the ambiguities and challenges they encounter with regard to lifestyle-related practices.

Finally, further research should also investigate the perspectives present in the social environment of parents—for instance, those expressed by grandparents, in order to gain insights in their sociocultural beliefs and behaviors regarding lifestyle-related practices. 

## Figures and Tables

**Table 1:** Characteristics of the respondents who participated in the study (total *N* = 38).

Female, *n (*%*)*	36 (*94.7*)
**Number pregnant at time of interview, *n* (%)**	3 (*7.9*)
**Age in years, mean (*range*)**	31.2 (*22–41*)
**Education level, *n* (%)**	
Low	3 (*7.9*)
Middle	10 (*26.3*)
High	17 (*44.7*)
Missing data	8 (*21.1*)
**Ethnicity, *n* (%)**	
Turkish	12 (*31.6*)
Moroccan	8 (*21.1*)
Dutch	6 (*15.7*)
Other ethnicities	12 (*31.6*)
**Living with, *n* (%)**	
Extended family	3 (*7.9*)
Nuclear family	35 (*92.1*)
**Number of children, *n* (%)**	
1	21 (*55.3*)
2	12 (*31.5*)
3	3 (*7.9*)
4	2 (*5.3*)
**Age of infant in months, mean (*range*)**	10.0 (*2–24*)
**Age categories of infants, *n* (%)**	
0–3 months	3 (*7.9*)
3–6 months	10 (*26.3*)
6–12 months	7 (*18.4*)
12–24 months	18 (*47.4*)

## Supplementary Materials

The following are available online at https://www.mdpi.com/1660-4601/17/16/5838/s1, Table S1. Characteristics of the respondents who participated in the study (total *N* = 38).

## Appendix A. Interview Guide with Parents about Lifestyle-Related Behaviors

**Food**
- How are you getting on with feeding your child? - Are you breastfeeding or bottle feeding? - What and how much does your child eat? - Why did you decide to breastfeed or bottle feed your child? - What was the transition from liquid to solid food like? - When did you start giving your child solid food? What did you give them? What kind of problems did you experience? - What kind of problems are you experiencing in relation to feeding? - What influences the type of food and drink you give your child?
**Sleep**
- How are things going with your child’s sleeping? - How does your child sleep? Do you think your child is getting enough sleep? - What do you do to get a better night’s sleep? - What kind of problems are you experiencing in relation to sleep?
**Physical activity**
- What kind of activities do you do with your child? - Do you think your child is active enough? - What kind of physical activities do you do with your child?
**Screen use**
- What do you think about young children using screens? - Do you use a screen? When or why do you use a screen? - How long should a child watch TV or use other electronic devices with a screen? - Do you put any limitations on screen use?
**Daily struggles**
- What problems do you face when it comes to caring for your child? - What type of information do you need about caring for your child? - Do you receive support in caring for your child? Who from and what kind of support? - How does your (social) environment or the neighborhood influence the healthy growth and development of your child? (barriers and facilitators) - What do you need to stimulate the healthy growth and development of your child?
**Perceived support**
- Who gives you support in caring for your child? - Who can you talk to if you want to discuss concerns about your child’s health or behaviors? (other parents, family, professionals)
**Health status**
- How would you describe a healthy child? - What role do food, sleep and physical activity play in the healthy growth of your child? - How would you assess the health status of your child?

## Appendix B. Statements or Questions for Discussion in Focus Group with Parents

**Health status (green cards)**
Statement. If parents eat healthy food at home, a child will adopt the same behavior. Statement. It is not a matter for concern if an infant/toddler is chubby; he/she will grow out of it. Statement. Watching TV is good for a child’s development. Statement. If children are overweight during infancy, they are usually overweight when they are older.
**Motherhood (pink cards)**
Question. How healthy do you feel? (How would you describe your own health?) Question. As a mother, how do you know what your infant needs? Statement. As a mother, you put the interests of your child first: your own health is less important. Question. How would you describe your maternal instinct?
**Fatherhood (blue cards)**
Question. In what way is your husband involved in the care of your child (ren)? Question. What is the role of a father during pregnancy? Question. How would you describe the influence of your husband on your child (ren)?
**Extended family (yellow cards)**
Statement. If my mother/mother-in-law gives my child something unhealthy to eat, I don’t say anything about it. Statement. I accept my mother’s advice on motherhood. Question. What is the role of the extended family (grandmothers/grandfathers/uncles/aunts) in your children’s health?
**Cultural influences (purple cards)**
Question. Does the advice you receive from Dutch healthcare providers fit in with your own culture? Question. What cultural differences are there when it comes to how you care for or bring up your infant?
