# Supplementary material for: Parental Perspectives and Experiences in Relation to Lifestyle-Related Practices in the First Two Years of a Child’s Life: A Qualitative Study in a Disadvantaged Neighborhood in The Netherlands
Source: Int J Environ Res Public Health. 2020 Aug 12;17(16):5838. doi: 10.3390/ijerph17165838 (PMC7460357; doi:10.3390/ijerph17165838)
Supplement: Supplementary file 1 [file ijerph-17-05838-s001.pdf]

International Journal of
*Environmental Research and Public Health*

MDPI

**Supplementary Materials:** The following are available online at www.mdpi.com/xxx/s1, Figure S1: title, Table S1: title, Video S1: title.

| **Table S1. Characteristics of the respondents who participated in the study (total N=38)** | |
|---|---|
| **Female, n *(%)*** | 36 *(94.7)* |
| **Number pregnant at time of interview, n *(%)*** | 3 *(7.9)* |
| **Age in years, mean *(range)*** | 31.2 *(22-41)* |
| **Education level, n *(%)*** | |
| Low | 3 *(7.9)* |
| Middle | 10 *(26.3)* |
| High | 17 *(44.7)* |
| Missing data | 8 *(21.1)* |
| **Ethnicity, n *(%)*** | |
| Turkish | 12 *(31.6)* |
| Moroccan | 8 *(21.1)* |
| Dutch | 6 *(15.7)* |
| Other ethnicities | 12 *(31.6)* |
| **Living with, n *(%)*** | |
| Extended family | 3 *(7.9)* |
| Nuclear family | 35 *(92.1)* |
| **Number of children, n *(%)*** | |
| 1 | 21 *(55.3)* |
| 2 | 12 *(31.5)* |
| 3 | 3 *(7.9)* |
| 4 | 2 *(5.3)* |
| **Age of infant in months, mean *(range)*** | 10.0 *(2-24)* |
| **Age categories of infants, n (%)** | |
| 0-3 months | 3 *(7.9)* |
| 3-6 months | 10 *(26.3)* |
| 6-12 months | 7 *(18.4)* |
| 12-24 months | 18 *(47.4)* |

© 2020 by the authors. Submitted for possible open access publication under the terms and conditions of the Creative Commons Attribution (CC BY) license (http://creativecommons.org/licenses/by/4.0/).
